# Sport, doping and male fertility

**DOI:** 10.1186/s12958-018-0435-x

**Published:** 2018-11-12

**Authors:** Andrea Sansone, Massimiliano Sansone, Diana Vaamonde, Paolo Sgrò, Ciro Salzano, Francesco Romanelli, Andrea Lenzi, Luigi Di Luigi

**Affiliations:** 1 Department of Experimental Medicine, Section of Medical Pathophysiology, Food Science and Endocrinology, Sapienza - University of Rome, Viale Regina Elena 324, 00161 Rome, Italy; 2 Morphological Sciences Department, School of Medicine, Universidad de Córdoba, Cordoba, Spain; 3 Department of Movement, Human and Health Sciences, Unit of Endocrinology, Università degli Studi di Roma “Foro Italico”, Largo Lauro de Bosis 15, 00135 Rome, Italy; 4 Dipartimento di Medicina Clinica e Chirurgia, Sezione di Endocrinologia, Università “Federico II” di Napoli, Naples, Italy

## Abstract

It is universally accepted that lifestyle interventions are the first step towards a good overall, reproductive and sexual health. Cessation of unhealthy habits, such as tobacco, alcohol and drug use, poor nutrition and sedentary behavior, is suggested in order to preserve/improve fertility in humans. However, the possible risks of physical exercise *per se* or sports on male fertility are less known. Being “fit” does not only improve the sense of well-being, but also has beneficial effects on general health: in fact physical exercise is by all means a low-cost, high-efficacy method for preventing or treating several conditions, ranging from purely physical (diabetes and obesity) to psychological (depression and anxiety), highly influencing male reproduction. If male sexual and reproductive health could be positively affected by a proper physical activity, inadequate bouts of strength – both excessive intensity and duration of exercise training – are more likely to have detrimental effects. In addition, the illicit use of prohibited drugs (i.e. doping) has reached pandemic proportions, and their actions, unfortunately very often underestimated by both amateur and professional athletes, are known to disrupt at different levels and throughout various mechanisms the male hypothalamic-pituitary-gonadal axis, resulting in hypogonadism and infertility.

## Introduction

Lifestyle interventions have been proven to be effective as the first step towards a good general health [1, 2] and even for maintaining adequate reproductive and sexual health [3, 4]. Physical exercise is widely considered to be one of the bases of a healthy lifestyle. However, contrary to popular belief, “being fit” does not necessarily mean “being healthy”. Solid evidence has suggested that adequate regular physical activity (i.e. physical exercise, sports, etc.) might have positive effects on cardiovascular, endocrine, metabolic and neurological status [5], whereas different forms of excessive and strenuous physical training often have deleterious effects on both general and reproductive health [6]. Furthermore, the widespread use of doping substances in general population and in both professional and non-professional athletes has become a worrying phenomenon in terms of health risk. Many people abuse hormones in order to provide better outcomes for their physical appearance, while – despite increasing efforts from the World Anti-Doping Agency (WADA) and from the different National Anti-Doping Organization (NADO) – athletes are constantly looking for new drugs to improve their sports performance.

**Fig. 1:**
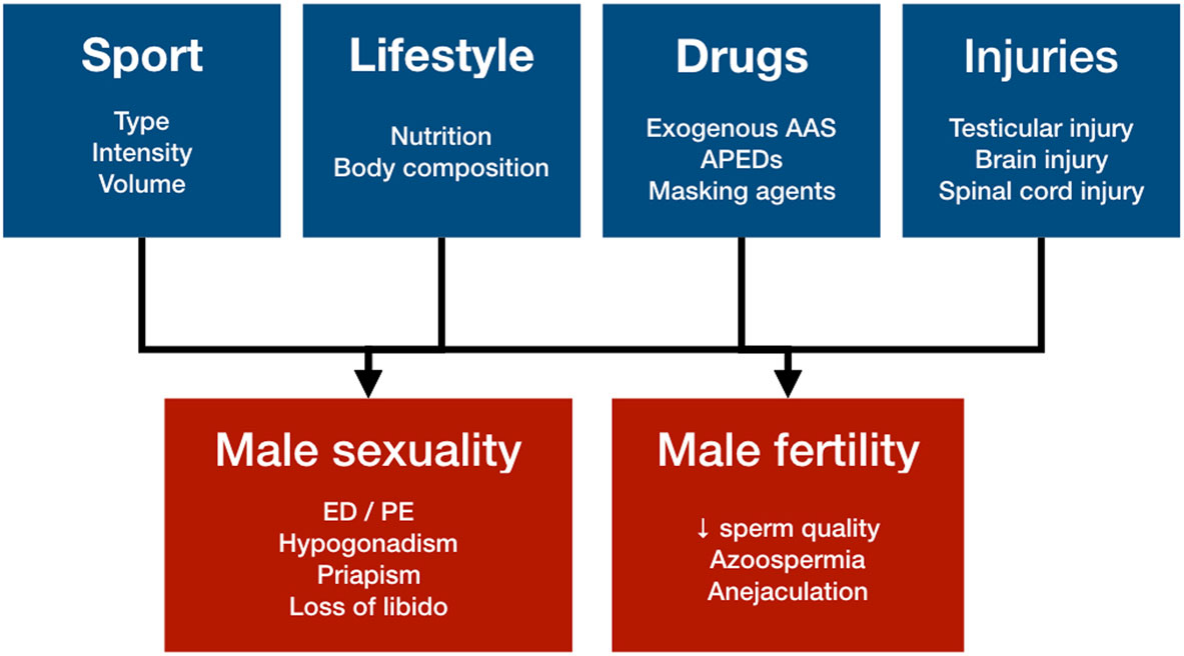
Disorders of male sexual and reproductive functions in athletes. Abbreviations: AAS, androgenic anabolic steroids; APEDs, appearance and performance-enhancing drugs; ED, erectile dysfunction; PE, premature ejaculation

Physical exercise and sports have been observed to have the potential for affecting human’s reproduction (i.e., reproduction-related sexual behavior and/or fertility), in both positive and negative ways. In males, sexual and spermatogenetic functions might be maintained or improved by adequate physical activity, whereas impairments in sexuality and/or in fertility have been observed [3, 7, 8] following excessive training and/or drug abuse (i.e. doping) (Fig. 1).

This review aims to address how physical exercise, sports and doping might either improve or impair reproductive capacity/fertility in males.

## Physical exercise, sports and the male hypothalamic-pituitary-gonadal (HPG) axis

The physiology of male reproduction is the result of fine tuning between several hormones involved in sexual function and spermatogenesis. For such purpose, the hypothalamus, pituitary and testes act together; therefore, it’s common practice to describe them as a single entity, namely the hypothalamic-pituitary-gonadal (HPG) axis. After puberty, pulsatile release of gonadotropin-releasing hormone (GnRH) from hypothalamic GnRH-secreting neurons stimulates production of luteinizing hormone (LH) and follicle-stimulating hormone (FSH) from secretory cells of the anterior pituitary; in males, FSH and LH act on the testes, stimulating spermatogenesis and testosterone production respectively. Secretion of GnRH is regulated by negative feedback mechanisms involving inhibin B, and both direct and indirect action from testosterone. Energy balance [9] dynamically regulates endogenous HPG axis, which in turn also regulates both exercise performance and success in competition [10]; unsurprisingly, both reduced androgen secretion and excessive levels of testosterone might have deleterious effects on performance as well as on general health [11].

### Physical exercise, sports and testosterone: The good

Testosterone is a widely-known determinant of muscle volume, strength, function and adaptation to exercise-related stress in humans (e.g. athletes, amateurs, and so forth), whether young or older [12–14]; however, its role goes further beyond, as testosterone might influence body composition, cognitive processes, glucose and protein metabolism, erythropoiesis and reproductive function in both athletes and non-athletes [15–20] (Table 1). Its effects are patent when employing it in replacement therapy in hypogonadal subjects [18–20].

**Table 1:** Physiological effects of testosterone on adaptation to physical exercise and sports performance in males. Edited from P Sgro and L Di Luigi [39]

Somatic Growth	Control of growth
	Epiphyseal cartilage closure
	Secondary sexual characteristics
	Somatic masculinization
Endocrine-metabolic system	Anti-cortisol effects *(metabolism, steroid receptor competition…)*
	Increased anaerobic glycolytic capacity
	Increased enzymes activity in mitochondria
	Increased phosphocreatine content
	Increased protein anabolism
	Increased sarco-tubular enzymes activity
	Inhibited stress related CRH-ACTH-Cortisol response
	Pro-insulin and insulin-like effects
	Reduced protein catabolism
	Stimulated erythropoiesis
	Synergic effects with growth hormone
Functional capacity	Increased aerobic and anaerobic capacity
	Cardiovascular efficiency
	Increased muscle strength and explosive strength
	Increased muscle adaptation to training
Body composition	Increased bone mineral density
	Increased muscle mass
	Male pattern muscle distribution
	Reduced fat mass
Central nervous system	Increased aggressiveness
	Increased dominance
	Increased inclination to command
	Increased motivation to compete
	Increased neuro-muscular conduction
	Increased visual-spatial capacities
	Reduced empathy
	Reduced negative reaction to external rapid stimuli and/or alarms
	Reduced perception of negative emotions
	Reduced sense of fatigue
Psycho-motor and sports capacity	Increased aggressiveness in competition
	Increased motivation to compete
	Increased resistance to fatigue
	Increased visual-spatial orientation during competition

Despite lacking evidence in regards to exact mechanisms involved, acute maximal and sub-maximal physical exercise may lead to a rapid increase in serum testosterone concentration, likely resulting in better adaptation and performance in both muscle activity and functional capacity during physical exercise [21]. Physical exercise and sports competition act as a stressor, and testosterone is likely acting as a homeostatic agent, together with the whole range of hormones secreted during or shortly after physical exercise [22]. Enhanced pituitary responsiveness, improved Leydig function, reduced testosterone clearance and changes in testicular blood flow have all been investigated [23–25] as possible causes; however, no definite evidence has been obtained so far, suggesting that a combination of mechanisms is the most likely explanation. The endocrine response to exercise related stress is related to various factors (e.g. type, intensity and duration of physical activity, etc.) [21, 26–28].

### Physical exercise, sports and testosterone: The bad

As previously stated, acute exercise often leads to a transient increase in testosterone levels; on the contrary, a marked decrease in testosterone levels has been suggested in athletes undergoing chronic exercise. Incorrectly executed exercise or poorly developed training programs elicit negative endocrine responses and physiological consequences [28]. The chronic exposure to excessive loads of endurance training may impair the function of the HPG axis, leading to significantly and persistently reduced basal (resting-state) free and total testosterone levels, often remaining within the physiological range [29–31], with no concurrent increase in LH levels. The resulting clinical and biochemical phenotype depicts the “exercise-hypogonadal male condition”, as defined by Hackney and colleagues [29, 30]; the pathophysiology of this condition have not been fully elucidated, although pituitary GnRH resistance, increased prolactin secretion and inhibitory effects on LH secretion from ghrein and leptin have all been considered as possible “triggers”. Retrospective studies have often shown a reduction of free and total testosterone concentrations in endurance-trained men, whereas prospective studies have often failed to provide definite results because of the varying features of the training period, the magnitude of training stimulus and the volume of training load employed [24].

To the present date, several authors have reported the effects of short-term and long-term intensive training: significantly decreased concentrations of total and free testosterone, as well as reduced FSH and LH secretion/pulsatility, and increased SHBG levels have been reported following high-intensity exercise [31, 32]. More recently, cortisol and testosterone dynamics following exhaustive endurance exercise have been described in endurance-trained males, suggesting that recovery from endurance exercise sessions at ventilatory threshold might require up to 72 h for free testosterone to return to baseline values [33]. Previous findings in high-altitude marathon runners [34] and professional cyclists [35] provide further confirmation with regards to the effects of different kinds of exercise on testosterone secretion and profile.

Whether testosterone suppression is the result of a physiological adaptation to stress or an undesirable side effect of excessive training is a matter still open to debate. The best treatment for exercise-induced hypogonadism is to reduce exercise load; however, constant monitoring of androgen status is suggested in all athletes undergoing strenuous training in order to promptly address any issues related to testosterone deficiency.

### Physical exercise, sports and testosterone: The ugly

Hormones, including testosterone, act on muscle strength, adaptation to exercise and recovery [26]: therefore, untreated male athletes with testosterone deficiency are likely to incur in specific risks for health and for their physiological adaptation and performance during exercise. Male athletes affected by untreated hypogonadism with hypo-testosteronemia (e.g. congenital hypogonadotropic hypogonadism, Klinefelter Syndrome, anorchia, and so forth) in association to worse results in terms of performance and adaptation to physical exercise and sports are more likely affected by increased risks for general health, ranging from osteoporotic fractures to cardiovascular accidents related to the combination of hypo-testosteronemia with high exercise-strain [6]. Testosterone deficiency is also, of course, an issue for all ages: hypogonadism is rarely overtly symptomatic in athletes [36] but may become a primary cause for concern in older subjects.

Given its properties, testosterone is in the WADA list of prohibited substances, and therefore athletes in need of treatment should obtain a Therapeutic Use Exemption (TUE) according to the WADA criteria by the respective NADO. To prevent misuse, both clinicians and athletes should respect the official therapeutic indications and authorized formulations/doses [37]. This procedure does not differ from others needed for other pathologies such as diabetes mellitus and adrenal insufficiency.

## Sports, sexual function and fertility in male athletes

### Sexual function

Reduced global physical exercise (i.e. sedentary behavior) is considered a risk factor for several chronic diseases and conditions, including sexual dysfunction in males [38, 39]; therefore, it should come as no surprise that adequate physical activity is fundamental to maintain or improve sexual health [39, 40] and that physical activity and the level of physical fitness are both associated to the quality of erectile function [39, 41, 42]. Better sexual health is closely associated with improvements in sexual activity and reproductive capacity. Lifestyle modifications, including physical exercise, are in fact suggested as a first-line treatment for erectile dysfunction [43], since exercise improves bioavailability of nitric oxide and increases the number of endothelial progenitor cells, while at the same time diminishing concentrations of markers of oxidative stress [44] and expression of TNF-α, IL-1-beta and IL-6, therefore improving endothelial function [45] and consequently reducing the risk for erectile dysfunction [46]. Two recent meta-analysis studies have proven the benefits of moderate, or moderate-to-vigorous healthy physical activity on erectile function, in both short-term and long-term interventions as well as in trials evaluating physical activity and exercise alone or in addition to usual care [47, 48]; however, suggesting that all kinds of physical exercise improve sexual health is misleading, as clearly proven in female athletes suffering from the “female athlete triad” – a syndrome featuring low energy availability, menstrual dysfunction, and low bone mineral density, frequently observed in a variety of sports [49]. Some analogies between female and male athletes have been described in regards to bone health and nutritional deficiencies, but there are also similarities concerning sexual dysfunctions in both sexes [50]. Male athletes suffering from exercise-induced hypogonadism are more likely to develop erectile dysfunction and ejaculatory disorders, as both conditions are also closely associated with reduced testosterone [51, 52]. Reduced sexual drive is among the classical symptoms of androgen deficiency [53], and testosterone supplementation is likely to improve sexual function in hypogonadal subjects [54]; however, a reduced exercise load is the suggested treatment in athletes suffering from exercise-induced hypogonadism [29], although unrealistic for athletes whose ultimate goal is to compete and win.

Premature ejaculation (PE) is the most common male sexual dysfunction, with an estimated prevalence ranging from 8 to 30% up to 22–38% in all age groups [52]. The pathogenesis of PE is complex, possibly involving psychological disturbances, alterations in hormonal status and lower urinary tract infections; however, as already mentioned in regards to erectile disorders, lifestyles seem to heavily influence orgasmic function. A negative association has been recently described between physical activity and PE, suggesting that PE is more likely to occur in patients reporting lower levels of physical exercise [55]: this preliminary finding, however, deserves attention since the significance of the association did not change despite correction for age, erectile dysfunction, alcohol use and body mass index.

### Fertility

Physically active subjects are more likely to have better sexual health, and are therefore more likely to have sexual activity [39]; however, despite the “need” to have sex in order to conceive, the issue of male fertility is a separate entity, requiring a completely different approach. Male fertility is remarkably affected by a variety of lifestyle factors, exerting either positive or negative effects on spermatogenesis [56]. Smoking, alcohol use, sedentary behavior are known risk factors for male infertility; however, how physical exercise may relate to male infertility is still unclear as contradictory results in different studies have been found to date [56, 57].

Different kinds of physical activity characterized by a different exercise load, might have different effects on sperm parameters [57]. The positive effects of an active lifestyle on spermatogenesis have been confirmed by comparing sperm parameters of physically active and sedentary men [58]; higher levels (in the normal range) of FSH, LH and testosterone have been described in physically active subjects compared to sedentary subjects. More recent reports have concluded that moderate training is associated with improvements in sperm DNA integrity and semen quality and with reduced expression of seminal markers of inflammation and oxidative stress [59–63]. However, once again, deleterious consequences for spermatogenesis have been repeatedly described following intensive exercise loads [31, 64, 65]; worse morphology and concentration have been described in triathletes compared to physically active subjects and water polo players, therefore suggesting that more strenuous feats might actually prove harmful to spermatogenesis and sperm DNA integrity, and even seminal antioxidant capacity [7, 57, 58, 66]. In some of the early years De Souza and colleagues already hypothesized a volume threshold to start observing reproductive alterations [65], besides the other findings already described both an increase in reactive oxygen species (ROS) production and a decrease in ROS scavengers have been described after 16 weeks of intensive cycling training in humans [64] as well as in animal models reflecting apoptotic events on the spermatogenic lineage [67, 68]. Cycling more than 5 h per week has been associated with a decline in both sperm concentration and motility [69].

Evidence would once again seem to suggest that moderate, healthy exercise is the key for a better overall health, whereas excessive loads of endurance training are more likely to induce worse outcomes, also in terms of fertility [8].

Besides volume and intensity, several factors might affect male fertility in athletes. Physical exercise is often suggested as a means for weight loss: in regards to male fertility, this has proven useful in improving sperm parameters [70]. However, adequate physical activity is only moderately useful for assisted reproduction techniques, as improvements in sperm parameters do not always mirror clinical outcomes (i.e. clinical pregnancy or live birth rates) [71]. Furthermore, as previously stated, excessive training might result in worse sperm parameters [69, 71], possibly negatively affecting the chances of successful treatment (Table 2). It should be taken into account that professional athletes perform higher absolute volumes of training at top-end intensity compared with amateur athletes; nevertheless, the same relative intensity elicits less physical stress on professional athletes compared to amateur ones [72, 73]. Training intensity should be considered different according to the athlete’s status (Tables 3, 4, 5). Furthermore, professional athletes may be more prone to evaluate training intensity according to parameters different from heart rate, which is affected by many variables (i.e stimulants, diet, hydration status, sports, temperature).

**Table 2:** Effects of sports and physical activity on human semen parameters: studies reporting effects of physical activity and sports on male fertility

Reference	Population studied	Results
Vaamonde et al. [7]	12 high-level triathletes after 2-weeks period of tapering (lowered training volume)	↑ DNA fragmentation ↑ round cells
Vaamonde et al. [57]	16 physically active subjects	Following more strenuous exercise: ↓ sperm count, ↓ morphology
	14 water polo players	
	15 “Ironman” triathletes	
Vaamonde et al. [66]	Case report - triathlete	↑ DNA fragmentation ↑ round cells ↓total antioxidant capacity
Vaamonde et al. [58]	16 physically active subjects	↑ sperm count and morphology, ↑ FSH, LH and T in physically active men
	15 sedentary men	
Maleki et al. [59]	280 men, randomized to moderate-intensity continuous training (MICT), high-intensity continuous training (HICT), high-intensity interval training (HIIT) or no-exercise	↑ semen quality and DNA integrity, ↓ markers of inflammation and oxidative stress in all exercise groups compared to no-exercise; best results for MICT compared to HICT and HIIT
Maleki et al. [60]	Semen samples from 56 elite athletes, 52 recreationally active men and 53 non-active men	↑ SOD, catalase and total antioxidant capacity and ↓ 8-Isoprostane, ROS and MDA in recreationally active men; ↓ SOD, catalase and total antioxidant capacity and ↑ 8-Isoprostane, ROS and MDA in elite athletes
Rosety et al. [62]	90 obese adults, randomized to either intervention group (45 men, 16-week aerobic training - treadmill) and control group (45 men).	↑ testosterone, sperm concentration, motility and morphology in intervention group
Maleki et al. [63]	433 Infertile men, randomly assigned to high-intensity exercise (*n* = 218) or control (*n* = 215) groups	↓ IL-6, TNF-α, ROS, MDA, and ↑ SOD, catalase, and total antioxidant capacity in high-exercise group
Maleki et al. [64]	24 non-professional male cyclists, undergoing 16 weeks of intensive training	↑ sperm ROS and MDA and ↓SOD, catalase and total antioxidant capacity, persisting up to 30 days post-exercise
Wise et al. [69]	4565 semen samples from 2261 men undergoing ART	↓ sperm concentration and motility in men bicycling ≥5 h/wk
Jozkow et al. [8]	177 young “lean, educated, and physically active” healthy males	↑ % immotile sperm in physical exercise (3rd – 4th quartile)
Maleki [61]	419 sedentary infertile men, randomized to either exercise (*n* = 210) or no exercise (*n* = 209)	↓ IL-1b, IL-6, IL-8, TNF-α, ROS, MDA, 8-isoprostane and ↑ sperm integrity, SOD, catalase, total antioxidant capacity and pregnancy rate in exercise group

**Table 3:** Levels of risk based on physical activity volume and intensity

Volume (hours/week) and intensity	Level of Risk
+ 30 h training or racing/weeks including moderate and high intensity [137]	Very high risk
20–30 h training or racing/weeks including moderate and high intensity [138]	High risk
10–20 h training or racing/weeks including moderate and high intensity [57, 139]	Medium risk
5–15 h training or racing/weeks not including moderate and high intensity [140–142]	Low risk
Less than 5 h training or racing/weeks not including moderate and high intensity [143, 144]	Very low risk

**Table 4:** Training Intensity for amateur athletes [143, 145–147]

Intensity	% Heart rate Reserve (HRR) or % oxygen uptake reserve (VO2R)	% Heart rate (HR) max	% maximum oxygen uptake (VO2max)
Very low	< 30	< 57	< 37
Low	30–39	57–63	37–45
Moderate	40–59	64–76	46–63
High	60–89	77–95	64–90
Very High (Elite training)	> 90	> 96	> 91

**Table 5:** Training intensity for elite athletes [73, 148, 149]

Intensity	% Heart rate (HR) max	% maximum oxygen uptake (VO_2_ max)	Ventilatory Threshold (VT)	Blood Lactate Levels (mM)	Lactate Threshold (LT)
Very low	54–73	50–65	< VT 1	< 1.2	< LT 1
Low	74–83	66–80		1.3–2.0	
Moderate	84–88	81–87	VT 1 – VT 2	2.1–3.6	LT 1 – LT 2
High	89–93	88–93		4.3–5.7	
Very High (Elite training)	> 94	94–100	> VT 2	> 5.8	> LT 2

#### Potential mechanisms involved in negative effects of physical activity on male fertility

Despite a solid body of evidence proving that excessive physical exercise might negatively affect male fertility, there is little knowledge concerning the potential mechanisms involved in the decrease of sperm quality. Exercise-induced hypogonadism is likely to occur in a minority of athletes; therefore, several hypotheses have been postulated in order to correctly address this issue. Any increase in scrotal temperature is likely to impair spermatogenesis by induction of germ cell death: mechanisms include autophagy, DNA damage and apoptosis [74]. Everyday clothing is supposed to have an effect on spermatogenesis [75]: tight undertrousers provide significantly more heat to testes compared to loose-fitting ones, a phenomenon seemingly attenuated by walking because of perigenital air circulation [76]. Whether more intense physical activity is associated with increased scrotal temperature is still a matter for debate [77, 78]. Exercise-induced hypoxia has been described in exercise training, as well as in aging [25]; testicular dysfunction observed in high-altitude exercise is possibly associated with reduced oxygen availability, and might impair male fertility resulting in reduced sperm concentration [79]. Similar reports have also addressed the effects of deep saturation dive on male fertility: negative effects on sperm motility and concentration were observed shortly after the dive and remained consistent up to 3 months later, suggesting that hyperbaric conditions are likely to impair spermatogenesis [80]. Pudendal nerve compression, repeated trauma on the pelvic floor and other morpho-functional alterations commonly observed in cycling have been considered possible causes of male infertility as well [81].

## Morpho-functional alterations and reproduction in male athletes

Some kinds of sports have been closely associated with male HPG morpho-functional alterations ultimately resulting in worse sexual function and/or reduced fertility. Brain and spinal cord injuries, as well as local trauma, are often cited as the most prevalent sports-related morpho-functional alterations possibly leading to erectile dysfunction and male infertility.

Traumatic brain injuries, as observed in football players [82] and in kickboxers [83], may lead to long-term impairment of pituitary secretion [84], most often acutely presenting as isolated GH deficiency [85] but possibly involving multiple axes during follow-up [86]. Causes of hypopituitarism following concussion are still unknown: there are a few hypotheses in these regards, suggesting a role of hemorrhage, edema, autoimmunity [87] or inflammatory and hypoxic state [85].

Pudendal nerve compression due to cycling is another complex, interesting phenomenon often resulting in urogenital complications [81]. Either because of mechanical pressure, transient hypoxemia, or arterial insufficiency, ischemic neuropathy is likely to occur in cyclists; this might provide an explanation to the increased odds ratio for erectile dysfunction in people cycling more than 3 h per week [88]. Cycling has also been associated with chronic prostatitis, a condition affecting both sexual and reproductive health [78, 81]. High-flow priapism is similarly more prevalent in cyclists: exercise-induced vascular trauma resulting in arterial-venous shunt is the most common etiology for this condition [81].

Increased abdominal pressure supposedly aggravates the development of varicocele in males. Rigano et al. reported significantly increased prevalence of higher grades of varicocele among athletes compared to non-athletes, with more severe forms affecting those training more than 6 h per week [89]. Physical activity might also be an aggravating factor for athletes with varicocele: despite there being no statistically significant difference in sperm parameters between healthy athletes and controls, varicocele seems to be associated with worse outcomes in athletes compared to non-athletes in terms of sperm morphology [90]. All athletes with varicocele should be clinically monitored, and treatment should be proposed if needed in order to preserve fertility and guarantee safe sport participation. More recently, Zampieri and Dall’Agnola suggested that sports practice might facilitate progression to clinical varicocele only in subjects affected by subclinical forms of varicocele [91].

Sports-related spinal cord injuries (SCIs) are uncommon outcomes of physical activity, although their rate is remarkably higher in some kinds of sports, such as rugby, diving and horseback riding [92]. The incidence of SCIs has dramatically reduced in the last decades; however, the devastating effect on the involved athletes’ quality of life has often rekindled media attention in these regards. Spinal trauma may result in different clinical syndromes based on the location of the lesion and on subsequent secondary events, such as hemorrhage and edema [93]. SCIs most frequently occur in young, male athletes as compared to female athletes [94] and impair the ability to obtain erection and to achieve ejaculation. Parasympathetic fibers originating from S2-S4 and entering the corpora cavernosa are needed for erectile function; patients with SCIs often lose psychogenic erections but may maintain reflexogenic erections, although this rarely allows for intercourse [94]. Both sympathetic fibers from segments T10-L2 and somatic fibers from segments S2-S4 are involved in the ejaculatory reflex; more in detail, fibers from the segments T11-L2 transmit a signal via the hypogastric nerve plexus ultimately resulting in emission [95]. Semen samples might still be collected, either through penile vibratory stimulation or via electro-ejaculation [96], and might often represent the only chance for fatherhood for these subjects.

Last, but not least, the issue of pelvic trauma deserves attention: in fact, more than half of testicular injuries occur during physical activity, most often as a result of blunt force trauma. In the most severe circumstances the resulting testicular damage might lead to primary hypogonadism; furthermore, the creation of anti-sperm autoantibodies might also affect male fertility [97]. Testicular torsion usually presents without any previous traumatic injuries to the scrotum, with only 4–8% of cases resulting from trauma [98]; however, some kinds of physical activity, most notably cycling, are linked to an increased risk of testicular torsion [81]. Potential mechanisms resulting in testicular torsion are the repeated up-and-down movements of the legs, contraction of the cremasteric muscles and exaggerated cremasteric reflex [81]. Blunt force trauma applied to the perineum might also result in penile injuries, although much less commonly than testicular injuries [99].

## Doping, sexual function and fertility in male athletes

The “doping epidemics” has crossed the boundaries of elite sports, and now appeals to a wide audience of subjects – from medal-winning athletes aiming to excel in competitions, to amateurs aiming to improve their looks without effort, “appearance and performance-enhancing drugs” (APEDs) use is widespread and seemingly increasing in recent years [100, 101]. The WADA has been actively involved in doping control, and several athletes have been sanctioned for doping use; however, the issue with APEDs lies not only in the effects on performance, but also in the possible – and often misjudged – side effects associated with their use (Table 6). Furthermore, many supplements have hormone-related effects despite being advertised as “natural compounds” [102], or might affect the endocrine milieu through indirect pathways [103], possibly by tampering with the “biological clock” and therefore acting as endocrine disruptors.

**Table 6:** Doping agents and their effects on male sexual and reproductive health

Substance(s)	Used:	Effects on…			
		Sexual desire	Erectile function	Ejaculatory function	Male fertility
Androgenic anabolic steroids	– for ergogenic and anabolic actions – for effects on CNS (↑aggressiveness, ↑ competitiveness…)	↑ or ↓	↓	↓ (delayed ejaculation, anejaculation)	↓
β-blockers	– to ↓ anxiety – to ↓ tremors – to ↓ heart rate		↓	↓ (delayed ejaculation, anejaculation)	
Diuretics	… as masking agents … for bodybuilding		↓	↓ (delayed ejaculation, anejaculation)	
Amphetamine, stimulants	… for effects on CNS (↑aggressiveness, ↑ competitiveness…) … for (unproven) ergogenic properties	↑ or ↓	↓	↑ or ↓ (premature delayed or ejaculation, anejaculation)	↓

### Testosterone and androgenic anabolic steroids

It is widely accepted that administration of exogenous testosterone and/or of other androgenic anabolic steroids (AAS) exerts a suppressive effect on the HPG axis [104]. Sexual and reproductive health are often impaired as a result of AAS-induced hypogonadism [39, 104]. Erectile dysfunction might be the first symptom of prolonged AAS abuse for subjects who are not interested in their fertility: clinicians should therefore pay attention to signs of AAS-induced hypogonadism during clinical assessment. Erectile dysfunction often occurs during the “post-cycle” period in abusers, when serum testosterone reaches its minimum, and is often associated with use of some AAS, most notably nandrolone [105].

Up to 2% of cases of male infertility can be explained by AAS abuse, whereas the vast majority of behavior-related infertility in female athletes is the result of excessive exercise [106]. Unsurprisingly, low levels of FSH are closely associated with reduced sperm count; clinically, prolonged abuse of AAS is likely to induce testicular shrinkage because of tubular atrophy, due to HPG axis suppression. Studies in animal models have also suggested possible effects of AAS on male germ line apoptosis, as observed via TUNEL, caspase-3 assay and transmission electron microscopy [107, 108]. Compared to erectile function, reproductive function is less likely to be permanently impaired by AAS: for most people spontaneous resolution of AAS-induced oligozoospermia and azoospermia has been observed in less than a year after discontinuation of APEDs [109].

AAS suppress intra-testicular testosterone production by inhibition of the HPG axis [110]: therefore, selective estrogen receptor modulators (SERMs) and gonadotropins have been used in order to quicken recovery following AAS cessation [111, 112], but individual response to treatment is highly variable and therefore difficult to assess. Several factors complicate the assessment of the effects of AAS on reproductive and sexual function, such as multi-drug regimens “stacking” several molecules [113] and depot effects [114]: for the same reasons, recovery may take a long time, even 12–24 months following prompt discontinuation of APEDs. More severe effects might occur in patients abusing AAS in puberty, as changes in the HPG axis in this time frame might lead to long-lasting damage on sexual health and development [115, 116]. In these regards, several treatments have been proposed for AAS-induced hypogonadism: SERMs such as clomiphene citrate 25 mg on alternate days have been successfully used for management of low testosterone, possibly after a 4-week tapered course of testosterone replacement therapy for more severe cases [105]. Should testosterone levels fail to rise despite treatment, primary testicular failure should be suspected and recovery is limited. Recent reports on hCG-based combined treatment seem to suggest that the vast majority of cases of testosterone-related azoospermia or oligozoospermia would benefit from treatment, with no significant difference in regards to supplemental therapies [117]. Another issue lies in the frequent use of multi-drug regimens [113]: athletes often “mix” AAS and other substances, most notably hCG, in order to reduce the suppression on the HPG axis or in hopes of synergies between treatments. Little evidence has been published in these regards, but reports suggest that conjoined administration of hCG and AAS impairs fertility [118], similarly to what has been described in AAS-only abusers. In addition, we highlight that it is not known if and how the large use and abuse of not prohibited substances (e.g. supplements, ergogenic aids and drugs) in athletes (i.e. not considered doping), often associated to the assumption of prohibited substances (i.e. doping), could influence the reproductive axis [103, 119–121].

A question largely left unanswered is whether administration of AAS during puberty is likely to impair spermatogenesis: reports from over-tall boys treated with high doses of testosterone seem to suggest that recovery time is not different from non-treated subjects [104], but evidence is inconsistent and caution is therefore advised.

### Non-hormonal drugs used for performance and appearance enhancement

Despite being significantly less common than AAS, several substances have been used in different sports in order to enhance performance; however, negative effects of these drugs have been reported concerning sexual and reproductive function. Beta-blockers, often used in order to reduce anxiety and tremors in precision sports, are likely to worsen erectile function [122]; furthermore, in vitro studies have reported inhibitory effects of beta-blockers on smooth muscle in reproductive tracts, possibly resulting in delayed ejaculation or, in the most severe cases, anejaculation [123]. In regards to cardiovascular treatments, diuretics, most notably thiazides, might impair erectile function [124]: these drugs are sometimes used by athletes in order as masking agents for concomitant treatments [125], but use of these drugs by bodybuilders aiming to improve their physical appearance is an increasingly worrying phenomenon [126] and their possible side effects might be amplified by coexisting eating disorders [127]. Amphetamines and stimulants – including some over the counter treatments, such as pseudoephedrine [128] – are used for their effects on the central nervous system [129]: by enhancing reflexes, increasing aggressiveness and (possibly) having ergogenic effects, these drugs are ideal candidates as doping agents in sports involving short bursts of speed or strength [130]. Among their side effects, these substances might both enhance or worsen erectile function, sexual drive and ejaculatory latency [131]; in animal models, testicular damage resulting in impaired fertility has been described in rats treated with amphetamine, suggesting a negative effect on spermatogenesis as well [132]. Carnitine, a compound with reported antioxidant properties, is often used as an APED; while some effects on sperm parameters have been reported in several in vivo studies using less than 3 g/day, there is no evidence on the effects of high carnitine intake on male fertility [133]. Similarly, several other compounds are often used for performance enhancing purposes, such as amino acids and soy or milk proteins. Some authors have reported negative effects of soy proteins on male fertility [134], although other studies have suggested otherwise [135]; likewise, creatine and other compounds commonly used by athletes, have not been adequately investigated in regards to their possible effects on spermatogenesis and fertility [136].

## Conclusions

Physical exercise and sports may exert both beneficial and deleterious effects on male sexual and reproductive health, depending on the imposed demands of exercise mode itself. Physical exercise is usually a valid tool in prevention of non-communicable diseases, such as heart disease and obesity, and is therefore generally suggested to all patients as a first-line therapy for a variety of conditions. However, while adequate physical exercise is key to a healthy lifestyle, excessive intensity or volume of training often lead to negative, undesired effects, such as infertility and sexual dysfunction. Furthermore, physical injuries resulting from sports, such as head trauma or spinal cord injuries, might have negative effects on general health, as well as sexual function. In order to identify whether reduced loads of exercise should be suggested in order to reduce the burden on male sexual and reproductive functions, assessment by a trained specialist is recommended for all athletes, whether professional or amateurs. Nevertheless, one must be aware that, especially in the case of professional athletes, it may be unrealistic to modify their training loads. Moreover, doping has a negative impact on male reproductive function, not only on semen quality but having an effect on the endocrine regulatory axis as well as the testes and accessory ducts, and sexual performance as evidenced in both human and animal model studies. This effect has been observed to revert in approximately 1 year upon cessation of substance abuse, except in extreme cases. Some studies assessing the impact of AAS abuse reflect that athletes may later on regret taking them. Therefore, it seems logical that clear and ample information to athletes about possible risks of exercise and AAS abuse is a must.
